# Prenatal treatment for opioid dependency: observations from a large inner-city clinic

**DOI:** 10.1186/s13722-016-0070-9

**Published:** 2017-01-13

**Authors:** Kelley Saia, Sarah M. Bagley, Elisha M. Wachman, Payal P. Patel, Marisa D. Nadas, Susan B. Brogly

**Affiliations:** 1 Department of Obstetrics and Gynecology, Boston University School of Medicine, 85 East Concord Street, Boston, MA 02118 USA; 2 Section of General Internal Medicine, Boston University School of Medicine, Boston, MA USA; 3 Department of Pediatrics, Boston University School of Medicine, Boston, MA USA; 4 Harvard Vanguard Medical Associates, Newton Wellesley Hospital, Newton, MA USA; 5 Department of Obstetrics and Gynecology, Albert Einstein College of Medicine, Bronx, NY USA; 6 Department of Surgery, Queen’s University, Kingston, Canada

## Abstract

**Background:**

The objective of this study was to review changes in the prevalence of opioid use disorder in pregnancy, and to describe the prenatal care and neonatal outcomes following the implementation of buprenorphine treatment at a large US obstetrical clinic during the on-going opioid epidemic.

**Methods:**

We conducted a retrospective cohort study of 310 women (332 pregnancies) with opioid use disorders and their neonates delivered between June 2006 and December 2010 at an obstetrical clinic in the US. Trends in patient volume, characteristics and outcomes by calendar year were assessed using the Cochran–Armitage test and linear regression.

**Results:**

There was an almost two-fold increase in the volume of pregnant women treated annually from 2006 through 2010. Most women were treated with methadone (74%), with buprenorphine becoming more common over calendar time: 3.0% in 2006 to 41% in 2010. The mean dose of buprenorphine at delivery was: 11.4 mg in 2007, 14.1 mg in 2008, 14.1 mg in 2009, and 16.8 mg in 2010; an average increase of 2.1 mg year. There were no differences in mean methadone dose over time. From 2006 to 2010 there were increases in the prevalence of prescribed concomitant psychotropic medications and vaginal deliveries, and in the proportion of neonates treated pharmacologically for neonatal abstinence syndrome (NAS). NAS pharmacologic management also varied by calendar year with more use of neonatal morphine and clonidine in later years.

**Conclusions:**

The number of mother–infant pairs increased significantly from 2006 to 2010 and the clinical characteristics of these patients changed over time. Our experience reflects the rising increase in opioid use disorders in pregnancy and NAS, mandating the need for expansion of comprehensive prenatal care options for these women and their children.

## Background

Opioid use disorders affect a rising number of women of childbearing age [1]. From 2000 to 2009, opioid use among US pregnant women increased from 1.19 per 1000 hospital births to 5.63 per 1000 hospital births per year [2]. National attention has been given to the rising incidence of Neonatal Abstinence Syndrome (NAS) in the US, which is associated with the rise in opioid use disorders in pregnancy. NAS is a constellation of signs and symptoms of withdrawal from in utero exposure to opioids and other substances. NAS incidence increased five-fold between 2000 and 2012, currently accounting for 3% of admissions to neonatal intensive care units (NICUs) in the US [3]. Reducing the incidence of NAS begins with prevention of opioid use disorders during the childbearing years. Improving maternal and neonatal outcomes for these dyads requires expanded access to opioid treatment programs for pregnant women. Recognizing the barriers to care such as socioeconomic challenges, potential legal consequences, intimate partner violence and psychiatric comorbidities is paramount to providing effective and accessible treatment options for this population [4].

The introduction of office based prescribing of buprenorphine has increased access to opioid use disorder treatment in the US and allows for integration of opioid agonist treatment in prenatal clinic settings [5]. Historically, opioid agonist treatment with methadone was the gold standard for managing opioid use disorders in pregnant women. Based on geographic location, however, many women do not have access to a methadone treatment facility in the US [6, 7]. In addition, most obstetricians have limited training in addiction medicine or substance use disorder screening methods and are unable to provide comprehensive care for these patients [8, 9]. Even in areas where such infrastructure exists, many women are resistant to starting methadone citing the stigma of methadone treatment and the restrictions of daily dosing as the major barriers [7]. The Project Recovery-Empowerment-Social Services-Prenatal Care-Education-Community-Treatment (RESPECT), Substance Use Disorder Treatment in Pregnancy Clinic, a multidisciplinary program based at an urban, academic center was developed in response to these issues. The objectives of this study are to describe changes at our center in the prevalence of opioid use disorder in pregnancy, the delivery of prenatal care, and neonatal outcomes following the implementation of buprenorphine treatment in 2006.

## Methods

### Setting and participants

We conducted a retrospective cohort study of pregnant women with opioid use disorder and their neonates delivered at Boston Medical Center from June 2006—when buprenorphine treatment began in our Project RESPECT clinic—through December 2010. Project RESPECT is a multidisciplinary treatment team at Boston Medical Center consisting of three buprenorphine waivered obstetric providers, a psychiatrist, an addiction psychiatry nurse practitioner, and a licensed independent clinical social worker. Boston Medical Center is an urban safety net hospital with a labor and delivery unit, high-risk maternal fetal medicine in-patient service, level three NICU and a pediatric in-patient ward for infants with NAS. Project RESPECT operates in conjunction with local and regional methadone clinics, counseling centers, and residential treatment programs for pregnant women.

Maternal criteria for opioid agonist therapy at Project RESPECT included being 18 years of age or older, DSM IV diagnosis of substance dependence for opioids, laboratory and/or radiographic documentation of pregnancy, and voluntary consent to engage in Project RESPECT’s comprehensive treatment program. All Project RESPECT patients treated with opioid agonist treatment (methadone or buprenorphine) were followed by the same specialized team. The type of opioid agonist treatment initiated was selected considering the following factors: patient choice, treatment history, disease severity and medical and psychiatric comorbidities.

Our practice protocol recommends methadone for women with more severe opioid use disorder, women with intolerance to buprenorphine, or for whom buprenorphine is medically contraindicated. In general, women for whom previous buprenorphine treatment was non-efficacious, who had a history within the last 6 months of buprenorphine diversion, who were unable to present for weekly to bi-weekly office visits, or who were unwilling to engage in independent relapse prevention counseling were offered methadone. All patients were scheduled for a prenatal care and relapse prevention visit every 1–3 weeks from initiation of care until delivery. Observed urine drug tests were done at every prenatal visit and on admission to labor and delivery. If an appointment was missed, patients were contacted and requested to come in for a urine drug test within 48 h.

### Study data

Study data were obtained from electronic medical records and included maternal age, total number of prenatal visits, urine drug test results, gestational age at treatment initiation, methadone or buprenorphine dose at treatment initiation and at delivery, concomitant medication use, smoking during pregnancy, gestational age at delivery, mode of delivery, anesthesia use during delivery, infant birth weight, neonatal abstinence score using a modified Finnegan scale, amount and duration of NAS opioid treatment and length of neonatal hospital stay [10]. Neonatal treatment with opioid therapy was initiated if the neonate had three consecutive modified Finnegan scores ≥8 or two consecutive scores ≥12. Neonates delivered June 2006 through mid-January 2009 had first-line treatment with diluted tincture of opium (DT0) of 0.4 mg morphine/mL, and neonates delivered mid-January 2009 through December 2010 had first-line treatment with neonatal morphine solution. Results were converted to total morphine in equivalents over the course of the hospitalization. Morphine dosing was based on both severities of scores and neonatal birth weight. Adjunctive therapy with phenobarbital or clonidine was added for infants who continued to have scores >8 despite maximum dosing of DTO or morphine. Infants were weaned off DTO, morphine, and/or clonidine as inpatients and monitored for 24–48 h prior to discharge. Phenobarbital was weaned in the outpatient setting. This study was approved by the Boston University Medical Center Institutional Review Board.

### Statistical methods

Trends in the treatment of mother–neonate pairs by calendar year were assessed using the Cochran–Armitage test for categorical variables and linear regression for continuous variables. For the linear trend model, year of birth was standardized to have a mean of zero and a standard deviation of 1 because of the small range of values relative to the mean in the original distribution. Differences in binary maternal and infant characteristics by prenatal exposure (methadone or buprenorphine) and calendar year were estimated using generalized linear models (log-link function) because the odds ratio overestimates the risk ratio given the high risk of our outcomes [11]. Linear regression was used to estimate differences in continuous outcomes. Women with >1 pregnancy were included for each pregnancy. Five women initiated therapy with buprenorphine and later were switched to methadone; these mother–neonate pairs are described, but were not included in our statistical models. One twin from each twin birth was included; analyses were run including and excluding the twin and results were unchanged. We did not account for the clustering in the small number of women (N = 20) who delivered infants from >1 pregnancy. Statistical analyses were carried out with SAS 9.3.

## Results

A total of 316 pregnant opioid dependent women presented for care during the study period: 296 women had one pregnancy, 18 had two pregnancies, and two had three pregnancies; there were six sets of twins. Of the 338 pregnancies, 6 resulted in intrauterine fetal demise or still birth and the remaining were live births. The final study population included 332 mother neonate pairs from 332 pregnancies (including one twin from each of the six sets of twins) in 310 women.

The number of pregnant women with opioid use disorders rose from 2006 through 2010. There was an almost two-fold increase in the number of women treated annually from 2007 through 2010. Following the emergence of buprenorphine as a treatment option at our clinic in 2006, women treated with buprenorphine for opioid use disorder increased from 3% in 2006 to 41% in 2010. The delivery dose of buprenorphine increased on average by 2.1 mg per year from 2007 to 2010 but no such trend in the average methadone dose at delivery was found. The prevalence of prescribed concomitant psychotropic medications increased as well as the proportion of vaginal deliveries (Table 1). There was a slight decrease in the proportion of maternal urine drug tests positive for cocaine. The proportion of neonates treated pharmacologically for NAS increased over time.

**Table 1:** Maternal characteristics by calendar year for 332 pregnancies

Characteristic	2006 (N = 33)	2007 (N = 53)	2008 (N = 69)	2009 (N = 84)	2010 (N = 93)
Maternal age (years)	27.4 (6.0)	28.8 (5.8)	28.6 (4.7)	27.2 (4.8)	27.3 (4.7)
Gestational age at presentation for care (weeks)	18.6 (9.1)	17.0 (8.0)	18.7 (9.7)	15.3 (7.7)	16.4 (9.9)
Number of prenatal care visits	8.9 (4.9)	9.6 (4.6)	9.6 (5.0)	9.4 (4.4)	9.3 (4.3)
Initial daily dose of methadone (mg)	70.9 (35.1)	66.1 (36.4)	74.2 (49.2)	66.6 (34.0)	64.9 (29.4)
Daily dose of methadone at delivery (mg)	88.5 (36.8)	83.8 (40.4)	94.4 (52.7)	91.0 (40.8)	88.0 (35.1)
Initial daily dose of buprenorphine (mg)	–	11.6 (7.0)	10.1 (4.0)	13.2 (6.5)	12.2 (7.2)
Daily dose of buprenorphine at delivery (mg)	–	11.4 (8.4)	14.1 (6.4)	14.1 (6.1)	16.8 (8.4)
Prescribed psychiatric medications					
SSRIs	3 (9.1)	4 (7.6)	14 (20.3)	13 (15.5)	20 (21.5)
Benzodiazepines	5 (15.2)	10 (18.9)	15 (21.7)	15 (17.9)	20 (21.5)
Antipsychotics	0 (0)	4 (7.6)	4 (5.8)	4 (4.8)	7 (7.5)
Other	2 (6.1)	1 (1.9)	5 (7.3)	8 (9.5)	10 (10.8)
Smoked cigarettes	25	36	42	59	59
Hepatitis C infected	19	27	38	48	48
≥1 urine screen	31 (93.9)	50 (94.3)	69 (98.6)	79 (94.0)	88 (94.6)
≥1 positive urine screen	15 (48.4)	22 (44.0)	38 (55.9)	41 (51.9)	43 (48.9)
Urine screen positive for^a^					
Cocaine	10 (32.3)	11 (22.0)	16 (23.5)	18 (22.8)	16 (18.2)
Opioids	11 (35.5)	14 (28.0)	26 (38.2)	32 (40.5)	44 (38.6)
Benzodiazepines	1 (3.2)	4 (8.0)	9 (13.2)	8 (10.1)	3 (3.4)
Amphetamines	1 (3.2)	1 (2.0)	3 (4.4)	2 (2.5)	3 (3.4)
Vaginal delivery	16 (48.5)	27 (50.9)	38 (55.1)	53 (63.1)	62 (66.7)
Cesarean section	17 (55.5)	26 (49.1)	31 (44.9)	31 (36.9)	31 (33.3)
Anesthesia during delivery	25 (75.7)	48 (90.5)	60 (86.9)	76 (90.4)	81 (87.0)

Missing data: Initial daily dose of opioid agonist therapy, one women treated with buprenorphine in 2008, one women treated with buprenorphine in 2009, one women treated with buprenorphine in 2010, one women treated with methadone in 2006, two women treated with methadone in 2007, one woman treated with methadone in 2009; Gestational age at first prenatal visit, two women in 2006, six women in 2007, three women in 2008, five women in 2009, two women in 2010. Smoking, 6 women in 2006, 10 women in 2007, 16 women in 2008, 15 women in 2009, 18 women in 2010; Hepatitis C, 10 women in 2006, 14 women in 2007, 15 women in 2008, 17 women in 2009, 18 women in 2010. Anesthesia, 5 women in 2006, 2 women in 2007, 3 women in 2008, 3 women in 2009, 3 women in 2010

Mean ± standard deviation or number of pregnancies and percent

^a^Patients with the prescribed medication not counted as a positive test result

There were some notable differences in maternal characteristics by opioid agonist treatment type (Table 2). Women treated with buprenorphine versus methadone were more likely to deliver in later calendar years, to have on average three more prenatal care visits, and had a 15% increased likelihood of vaginal delivery. Buprenorphine treated women had a 26% lower risk of a positive urine screen for any illicit drug after treatment initiation, 76% lower risk of a positive cocaine screen and 31% lower risk of a positive opioid screen compared to those treated with methadone.

**Table 2:** Maternal characteristics by prenatal opioid agonist treatment for 332 pregnancies

Characteristic	Mean and standard deviation or number and proportion		Mean difference or risk ratio and 95% CI (adjusted for year of birth)	
	Buprenorphine (N = 82)	Methadone (N = 245)	Buprenorphine and methadone (N = 5)	Buprenorphine versus methadone
Maternal age (years)	28.1 ± 5.1	27.7 ± 5.1	27.2 ± 3.3	0.76 (−0.55, 2.07)
Year of delivery^a^				
2009–2010	60 (73.2)	114 (46.5)	3 (60.0)	1.57 (1.30, 1.90)
2006–2008	22 (26.8)	131 (53.5)	2 (40.0)	1.0
Number of fetuses				
Singleton	80 (97.6)	241 (98.4)	5 (100)	–
Twin	2 (2.4)	4 (1.6)	0 (0)	
Prenatal care	82 (100)	241 (0.99)	5 (100)	–
Gestational age at presentation for care (weeks)	16.4 ± 8.9	17.2 ± 8.9	11.8 ± 8.6	−0.16 (−2.46, 2.13)
Number of prenatal care visits	11.5 ± 4.7	8.7 ± 4.3	11.6 ± 2.2	3.06 (1.92, 4.20)
Initial daily dose of opioid agonist therapy (mg)	12.0 ± 6.5	68.3 ± 37.4	12.0 ± 4.0	–
Stopped agonist therapy during pregnancy				
Yes	2 (2.4)	0 (0)	0 (0)	–
No	80 (97.6)	245 (100)	5 (100)	
Daily dose of opioid agonist therapy at delivery (mg)	15.1 ± 7.5	89.3 ± 41.7	77.0 ± 28.9	–
Smoked cigarettes	54 (65.9)	164 (66.9)	4 (80.0)	–
Hepatitis C infected	32 (39.0)	134 (54.7)	4 (80.0)	–
Prescribed SSRIs	14 (17.1)	38 (15.5)	2 (40.0)	1.01 (0.57, 1.79) 1.0
Prescribed benzodiazepines	14 (17.1)	51 (20.8)	0 (0)	0.81 (0.47, 1.39) 1.0
Prescribed antipsychotics	1 (1.2)	18 (7.4)	0 (0)	0.15 (0.02, 1.12) 1.0
Other prescribed psychiatric medications	9 (11.0)	15 (6.1)	2 (40.0)	1.60 (0.72, 3.60) 1.0
Urine screen				
≥1	78 (95.1)	233 (95.1)	5 (100)	1.02 (0.95, 1.09)
None	4 (4.9)	12 (4.9)	0 (0)	1.0
Positive urine screen				
≥1	31 (39.7)	123 (52.8)	5 (100)	0.74 (0.55, 1.01)
None	47 (60.3)	220 (47.2)	0 (0)	1.0
Cocaine urine screen				
Positive	5 (6.4)	63 (27.0)	3 (60.0)	0.24 (0.10, 0.57)
Negative	73 (93.6)	170 (73.0)	2 (40.0)	1.0
Non-prescribed opioid urine screen				
Positive	22 (28.2)	90 (38.6)	5 (100)	0.69 (0.46, 1.02)
Negative	56 (71.8)	143 (61.4)	0 (0)	1.0
Non-prescribed benzodiazepines urine screen				
Positive	7 (9.0)	18 (7.7)	0 (0)	1.27 (0.54, 2.98)
Negative	71 (91.0)	215 (92.3)	5 (100)	1.0
Non-prescribed amphetamine urine screen				
Positive	3 (3.9)	7 (3.0)	0 (0)	1.34 (0.34, 5.31)
Negative	75 (96.1)	226 (97.0)	5 (100)	1.0
Mode of delivery				
Vaginal	55 (67.1)	138 (56.3)	3 (60.0)	1.15 (0.95, 1.39)
Cesarean section	27 (32.9)	107 (43.7)	2 (40.0)	1.0
Anesthesia during delivery				
Yes	76 (93.8)	209 (90.9)	5 (100)	1.03 (0.96, 1.11)
No	5 (6.2)	21 (9.1)	0 (0)	1.0

Missing data: Initial daily dose of opioid agonist therapy, three women treated with buprenorphine, six women treated with methadone; Gestational age at first prenatal visit, one woman treated with methadone. Number of prenatal care visits, two women treated with methadone; Smoking, 12 women treated with buprenorphine, 52 women treated with methadone, 1 woman treated with methadone and buprenorphine; Hepatitis C infection, 11 women treated with buprenorphine, 62 women treated with methadone, 1 woman treated with methadone and buprenorphine; Anesthesia, one women treated with buprenorphine, and 15 women treated with methadone

^a^Unadjusted result

Neonatal characteristics and NAS outcomes by calendar year are provided in Table 3. Following the changes in prenatal opioid agonist therapy at our site, the proportion of infant exposed to buprenorphine increased over time. The proportion of infants treated pharmacologically for NAS also increased from 76% in 2006 to 86% in 2010. The length of NAS treatment appears to have decreased following the replacement of DTO with morphine as a first line NAS treatment in 2009 despite a trend towards treatment starting earlier as year of birth increased. There was no discernable trend in the amount of morphine per birth weight by year of delivery. Mean gestational age at birth was stable over time and above 37 weeks, but the proportion of infants born preterm was overall high.

**Table 3:** Neonatal characteristics by year of birth

Characteristic	2006 (N = 33)	2007 (N = 53)	2008 (N = 69)	2009 (N = 84)	2010 (N = 93)
Prenatal opioid agonist therapy exposure					
Methadone	32 (97.0)	48 (90.6)	51 (79.3)	62 (73.8)	52 (55.9)
Buprenorphine	1 (3.3)	5 (9.4)	16 (23.2)	22 (26.2)	38 (40.9)
Methadone and buprenorphine	0 (0)	0 (0)	2 (2.9)	0 (0)	3 (3.2)
Length of hospitalization (days)	23.2 ± 10.3	24.2 ± 12.7	24.1 ± 12.9	21.9 ± 11.1	20.8 ± 10.9
Pharmacologically treated for NAS	25 (75.8)	43 (81.1)	62 (89.9)	72 (85.7)	80 (86.0)
Age at NAS treatment Initiation (days)	2.5 ± 2.1	2.4 ± 1.9	2.9 ± 3.1	2.0 ± 1.7	1.9 ± 1.4
First-line NAS treatment					
Morphine	0 (0)	0 (0)	1 (1.6)	67 (93.1)	80 (100.0)
DTO	25 (100)	43 (100)	61 (98.4)	5 (6.9)	0 (0)
Total morphine used to treat NAS, mg per kg birth weight^a^	6.0 ± 3.7	6.7 ± 6.2	3.6 ± 3.5	8.9 ± 8.4	6.4 ± 5.4
Additional NAS treatment with Phenobarbital	9 (36.0)	9 (20.9)	10 (16.1)	23 (31.9)	18 (22.5)
Clonidine	0 (0)	0 (0)	0 (0)	2 (2.8)	8 (10.0)
Length of NAS treatment (days)	20.6 ± 9.6	20.6 ± 10.2	19.5 ± 10.1	19.2 ± 9.5	17.5 ± 7.8
Peak Finnegan score among neonates treated for NAS	13.9 ± 2.8	13.3 ± 3.5	11.9 ± 2.6	12.9 ± 3.0	11.9 ± 2.5
Gestational age at birth (weeks)	37.9 ± 1.8	38.7 ± 2.2	38.0 ± 2.5	38.2 ± 2.5	38.7 ± 2.3
Preterm birth (<37 weeks)	7 (21.2)	6 (11.3)	17 (24.6)	16 (19.1)	12 (12.9)
Birth weight (g)	2895.8 ± 373.1	2786.6 ± 728.0	2807.9 ± 599.6	2898.5 ± 652.3	2974.2 ± 580.2

Missing data: Birth weight, one neonate; Length of stay, one neonate; Total mg of morphine per kg of birth weight: one neonate; Peak Finnegan Score, twelve neonates

*NAS* neonatal abstinence syndrome, *DTO* diluted tincture of opium

^a^Morphine dose equivalent determined for DTO treated neonates

## Discussion

We describe outcomes of pregnant women with opioid use disorders and their neonates treated at our Project RESPECT clinic from 2006 to 2010. Over the study period, the percentage of women treated with buprenorphine increased from 3 to 41%, possibly reflecting increases in patient demand, increased patient autonomy, and improved provider prescribing comfort. Buprenorphine treatment failure, defined as transition from buprenorphine to methadone during the pregnancy for non-adherence, did not show a corresponding increase. The increase in the average dose of buprenorphine prescribed over the study period may be the result of several factors including, an increase in provider prescribing comfort over time and an increase in addiction disease severity in those seeking buprenorphine treatment. Choosing the appropriate agonist medication for pregnant women with opioid use disorder is complex; multiple variables including patient preference, disease severity, psychiatric comorbidities, social supports and recovery resources must be considered.

There were important differences observed in the characteristics of women by treatment approach. Women treated with buprenorphine versus methadone attended more prenatal care visits and had fewer positive urine drug tests. In addition, these women were more likely to delivery vaginally, likely due to changes in operative delivery recommendations for hepatitis C positive women. After 2007, cesarean sections were no longer recommended to decrease vertical transmission of hepatitis C. From 2006 to 2010, our cesarean section rate decreased from 45.8 to 35%, with the largest number of buprenorphine patients delivering in the later calendar years.

Maternal severity of opioid dependence and other factors influenced our clinical prescribing, with buprenorphine typically being used in more stable pregnant women who do not need the structure of observed daily dosing [12]. Potential confounding by indication can occur in assessing the comparative outcomes of prenatal BMT versus MMT because maternal characteristics that might influence choice of prenatal treatment with BMT versus MMT likely also affect neonatal outcomes [13, 14]. Prior studies have suggested improved NAS and birth outcomes in neonates exposed to buprenorphine compared to methadone, including decreased NAS severity with shorter length of hospitalization, lower risk of NAS pharmacologic treatment, and higher gestational age at birth, birth weight, body length and head circumference [15–17]. Because of the potential for uncontrolled confounding in this and many studies published to date [18] we did not assess any causal relationships between prenatal exposure and infant outcomes. Further, the dynamic nature of clinical care in this patient population, including changes in both maternal treatment and in NAS treatment, can introduce misclassification bias in any causal assessments.

We observed changes in NAS care practices in our institution over the calendar years, including a shift from DTO to neonatal morphine solution as first-line treatment, and more clonidine use for adjunctive second-line treatment. These observed changes fit with the updated recommendations for NAS management over the past decade. Morphine and methadone are recommended by the American Academy of Pediatrics (AAP) as preferred agents over DTO [19, 20]. While the majority of institutions in the US use morphine, a recent single-center randomized control trial found that methadone was associated with shorter hospitalizations in comparison with morphine [21]. In addition, some institutions have started to transition infants home on methadone to complete weaning [22, 23]. Recent national trends in adjunctive medications for NAS favor clonidine as an acceptable option over phenobarbital [24, 25]. There is also a trend towards more emphasis on breastfeeding, rooming-in, and other non-pharmacologic care interventions to best manage NAS [18, 26]. Lastly, though the Finnegan scale is the current gold standard for NAS assessments, newer scales are under development [26]. Significant variability in NAS care remains and there is a need for more high quality clinical trials to best guide management.

Strengths of our study include being conducted at a single large clinic that is a leader in addiction medicine with standardized approaches for treating prenatal opioid use disorders and for assessing and treating NAS, and the largest sample size of opioid dependent mother–neonate pairs studied to date. Limitations of our study include the potential for error in chart abstraction and limited variables available clinically, especially potential confounders. Our institutional NAS clinical guidelines changed mid-way through the study period to use of neonatal morphine solution versus DTO as first-line therapy. Although DTO was converted to morphine equivalent dose, some differences in the pharmacology of these treatments may exist.

## Conclusions

The choice of opioid agonist treatment remains a complex issue, with the overriding goal of maintaining maternal stability throughout the pregnancy and the post-partum period to improve both maternal and neonatal outcomes. Future research is needed to evaluate whether maternal addiction severity and choice of opioid agonist treatment affect neonatal outcomes and maternal long-term recovery. Accessible prenatal care combined with opioid use disorder treatment for pregnant women in the US must be adopted to address the needs of this underserved and growing population. Boston Medical Center’s obstetric care and addiction medicine treatment clinic, Project RESPECT, demonstrates the feasibility of such a model in a large urban center. Reducing health care costs and improving the care for opioid-exposed newborns cannot be accomplished without parallel development and implementation of comprehensive care for opioid dependent pregnant women.
